# Long term results of patients with neovascular age-related macular degeneration switched from other anti-VEGF agents to intravitreal Aflibercept

**DOI:** 10.1186/s40942-022-00361-9

**Published:** 2022-02-10

**Authors:** Sean D. Adrean, Darren Knight, Siyang Chaili, Hema L. Ramkumar, Ash Pirouz, Scott Grant

**Affiliations:** 1 Retina Consultants of Orange County, 301 W. Bastanchury Ave #285, Fullerton, CA 92835 USA; 2 University of California San Diego, Shiley Eye Institute, La Jolla, CA 92093 USA

## Abstract

**Background:**

This study explores the long term anatomic and functional results of patients who were switched to intravitreal aflibercept injections (IAI) after being initially managed with other anti-VEGF agents for neovascular age-related macular degeneration (nAMD).

**Methods:**

Patients with nAMD were included if they started with another anti-VEGF agent and were switched to IAI. Subjects had at least 3 years of consistent therapy with IAI and at least 1 injection quarterly.

**Results:**

Eighty-eight patients had at least 3 years of treatment while 58 of those patients, had at least 4 years of IAI. Average treatment time with other anti-VEGF agents was 32 months prior to switching. Baseline best corrected vision (VA) was 59.4 letters (20/70 + 2). At time of switch, VA increased significantly to 66.7 letters (20/50 + 2). At 3 months after switch, VA increased significantly to 69.0 (20/40−) letters. After 3 years of consistent IAI, vision was 67.5 letters (20/40−2), and for those patients that completed 4 years of therapy, the average VA was 66.0 letters (20/50 + 2), with a gain of 6.6 letters over baseline vision. 32.1% of patients gained 3 or more lines of vision. Initial central macular thickness (CMT) was 369 µm, which improved to 347 µm at time of switch, and further improved at 3 months to 301 µm and was maintained over time.

**Conclusion:**

Patients switched to IAI can maintain vision over the long term. Patients treated on average for 5.7 years, had a visual gain of 8.1 letters after 3 years and 6.6 letters after 4 years of IAI therapy. CMT significantly improved following the switch and was maintained.

**Supplementary Information:**

The online version contains supplementary material available at 10.1186/s40942-022-00361-9.

## Background

Over the last 16 years, anti-vascular endothelial growth factors (anti-VEGF) therapy has revolutionized how retina specialists approach treatment of retinal disorders. Prior to anti-VEGF therapy, the treatment goal for neovascular age-related macular degeneration (nAMD) was to prevent significant vision loss. Today, many patients with nAMD are able to maintain or gain functional vision with anti-VEGF therapy.

In order to treat nAMD, retina specialists employ a variety of treatment strategies. The three main strategies are (1) fixed dosing using monthly or bimonthly injections, (2) PRN (Pro Re Nata) dosing typically beginning with a loading dose of 3 monthly injections, then monitoring when there is a “dry” macula and treating again with breakthrough exudation, and (3) treat and extend (TAE) protocols. In TAE protocols, 3 monthly loading doses are given, after which the time interval is extended by 1–2 weeks when a dry macula is achieved until, a time interval of 10–12 weeks between injections is reached [1, 2]. A variant of the TAE, is the Treat–Extend–Stop protocol, where treatment cessation is reached, when no disease activity is found over two 12-week intervals [3].

Seminal randomized clinical trials such as ANCHOR, MARINA, HARBOR, CATT and VIEW all demonstrated visual gain in the first 2 years mainly using a fixed interval dosing strategy [4–9]. The patients were then returned to office-based care and the extension trials were also published. Both the CATT 5 and the SEVEN-Up study, which were extension trials of CATT and ANCHOR and MARINA, respectively, showed that patients lost vision compared to baseline [10, 11]. Many of these patients were managed with a PRN strategy.

The VIEW extension trial showed positive visual gains after 4 years of therapy managed mainly by a fixed interval dosing protocol. We previously reported on our 8-year data using a Treat–Extend–Stop (TES) protocol and showed positive visual gains. We noticed that many patients had been switched to different anti-VEGF agents over the 8-year period [12]. Since there are multiple anti-VEGF agents available, when patients are determined to be non-responders or sub-optimal responders, the anti-VEGF agent may be changed to see if there is additional efficacy.

In this study, we describe the long term anatomic and functional outcomes in patients who were switched to intravitreal aflibercept injections (IAI) after being initially managed with other anti-VEGF agents. We analyzed visual outcomes at 3 and 4 years as well as the spectral domain optical coherence tomography (SD-OCT) data and number of injections given per year.

## Materials and methods

This retrospective study received IRB approval from IRBco. (2017-0050-RCOC) and the study adhered to the tenets set forth in the declaration of Helsinki. All of the information and data collected was in accordance with the Health Insurance Portability and Accountability Act (HIPAA). A database search of a retina only practice was performed from November 2005 until January 2019, to identify patients with neovascular Age-Related Macular Degeneration (nAMD). In order to be included in this study, patients required initial treatment with either ranibizumab (Lucentis; Genentech Pharmaceuticals, San Francisco, CA), bevacizumab (Avastin; Genentech Pharmaceuticals, San Francisco, CA), or both, followed by a switch to aflibercept (Eylea; Regeneron Pharmaceuticals, Tarrytown, NY) which was FDA approved November, 2011. We included patients that required treatment with a minimum of 1 IAI every 3 months for at least 3 years. There were no exclusion criteria based on initial vision, choroidal neovasclar lesion size, presence of subretinal hemorrhage, Retina Pigment Epithelium (RPE) tears, fibrosis or atrophy. Patients were excluded if they had irreversible causes of vision loss from non-retina related conditions, such as ischemic optic neuropathy or if they required surgical retina intervention, such as for massive subretinal hemorrhage.

For this study, data captured included visual acuity and central macular thickness (CMT) at baseline, at switch to IAI, at 3 months, and yearly to 3 years. If those patients had enough data for 4 years of therapy, that data was also collected. VA data was converted to Early Treatment Diabetic Retinopathy Study (ETDRS) letter equivalents for analysis.

**Fig. 1:**
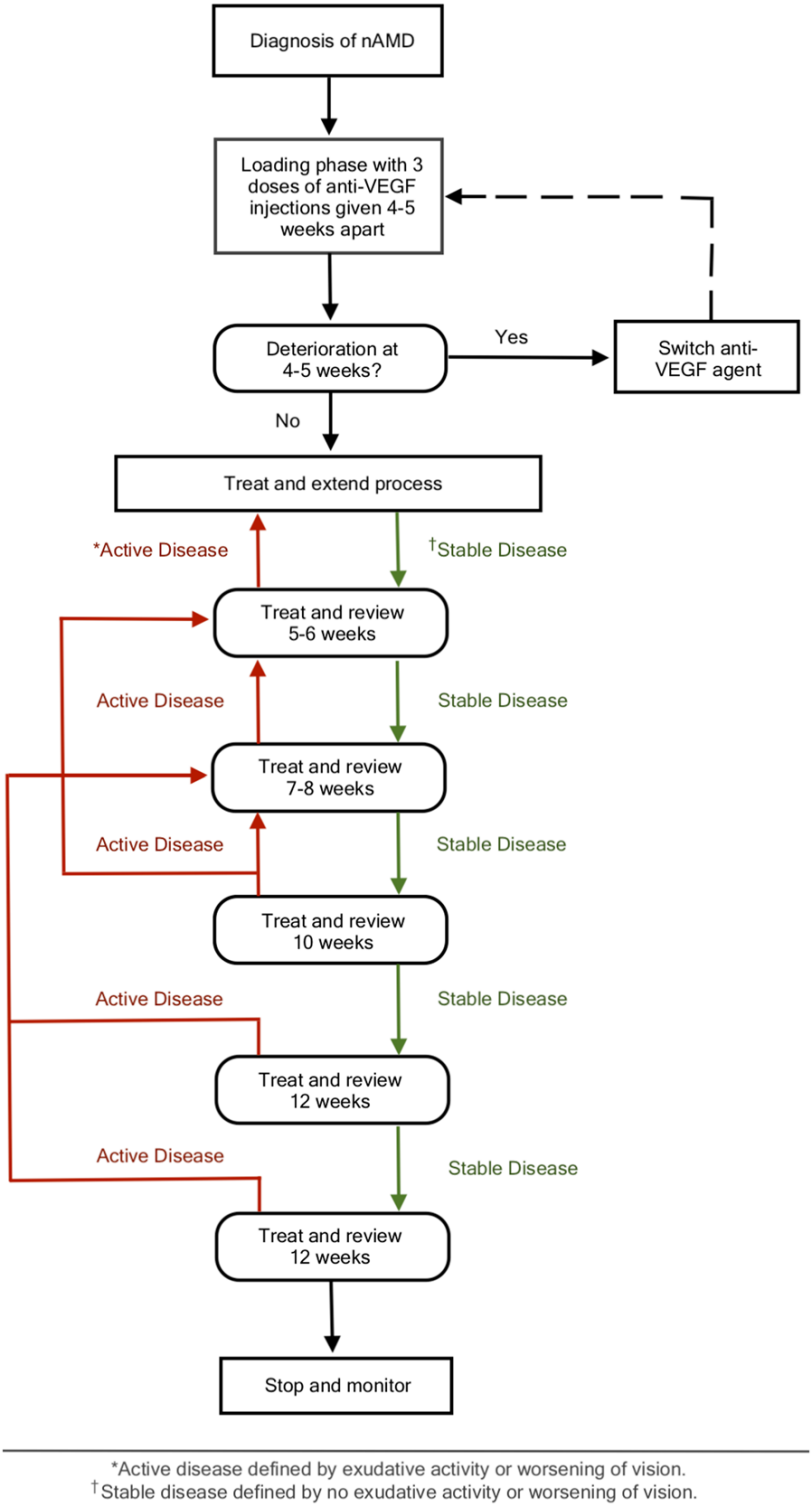
Flow diagram of treat–extend–stop protocol

Patients were treated using the TES protocol, a variant of the treat-and-extend regimen (Fig. 1). TES treatment protocol required 3 initial injections at 4 to 6 week time intervals. Upon clinical examination and on OCT, dosing interval was extended by 1 to 2 weeks in patients with a “dry” macula until a 12-week time interval was reached. If fluid or worsening of vision was detected, the dosing interval was reduced. After successful management of the choroidal neovascularization (CNV) with shorter intervals, patients were allowed to increase intervals by 1 week for the next 2 to 4 injections. Typically, if the patients were on a 4–6 week dosing interval with increasing exudation and fluid on SD-OCT, the patients were deemed to be treatment failures or suboptimal responders, and the anti-VEGF agent would be changed. In patients with good response without worsening vision but exhibiting presence of mild fluid during the initial 4 to 6 week treatment interval, the same interval was maintained. In patients with complete absence of fluid in the macula, dosing intervals were extended by 1 to 2 weeks. Extension of dosing intervals continued until 12 weeks was reached between visits.

The paired Student’s t-test was used for statistical analysis and a p value of < 0.05 considered to be statistically significant.

## Results

**Fig. 2:**
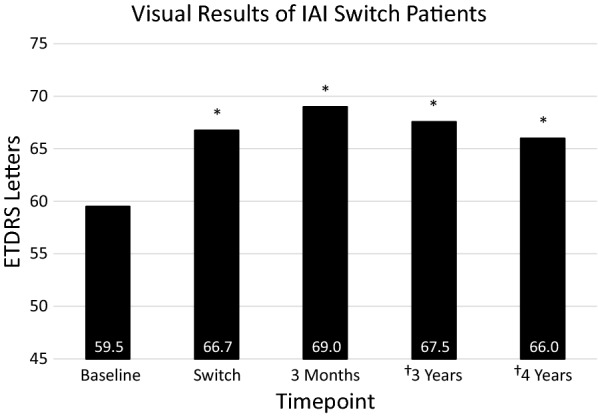
Visual acuity of IAI switch patients. Compared to baseline, VA at the time of switch significantly improved (*p < 0.0001) from 59.4 to 66.7 letters. From the time of switch to 3 months, VA also significantly improved (*p < 0.0001) from 66.4 to 69.0 letters. After 3 months, VA was significantly improved compared to baseline (*p < 0.0001). In Year 3, data is aggregated from 88 patients and in year 4, data is aggregated from 58 patients

A total of 1305 patients with nAMD were treated during the study period of November 2005 to January 2019. Eighty-eight patients met the inclusion criteria of initial anti-VEGF therapy with intravitreal ranibizumab or intravitreal bevacizumab and had at least 3 years of aflibercept. Fifty-eight of those patients had at least 4 years of therapy after switching. Patients on average had been treated for 32 months (range 1–96 months) with other anti-VEGF agents prior to the switch. The average VA at baseline was 59.4 ETDRS letters (20/70 + 2, Fig. 2). At the time of switch 25% of patients gained 3 lines of vision (Table 1), 2.3% of patients lost 3 lines of vision, and average VA increased to 66.4 ETDRS letters (20/50 + 2), a statistically significant gain of 7.3 ETDRS letters (p < 0.0001). At 3 months after the switch to IAI, 29.5% of patients gained 3 lines of vision, 2.3% of patients lost 3 lines of vision, and the average VA increased to 69.0 letters, a 9.5 letter gain from baseline, (20/40−). This gain was significant when compared to the vision at the time of switch (p < 0.0001). After 3 years of consistent IAI, 27.3% of patients gained 3 lines of vision, 10.2% of patients lost 3 lines of vision, and VA decreased to 67.5 letters (20/40−2). This was still a gain of 8.1 letters and was significantly better than baseline vision (p < 0.0001). For the 56 patients at 4 years of treatment, 32.1% gained 3 lines of vision, 10.7% lost 3 lines of vision, and the average VA was 66.0 letters (20/50 + 2). This was a gain of 6.9 letters from baseline (p = 0.0083), and similar to at time of switch (p = 0.44).

**Table 1:** Percent of patients gaining or losing 3 lines of vision

	At switch	3 months	3 years	4 years
3 lines gained	25.0%	29.5%	27.3%	32.1%
3 lines lost	2.3%	2.3%	10.2%	10.7%

Over 4 years after switching from other anti-VEGF agents to aflibercept, the number of 3-line gainers increased from 25.0 to 32.1% by 4 years, while the number of 3-line losers increased from 2.3 to 10.7% by 4 years

Following the switch to IAI, the average number of aflibercept injections at year 1 was 7.7 ± 2.2; year 2 was 6.5 ± 2.2; year 3 was 6.5 ± 2.3; and year 4 was 6.6 ± 2.7.

**Fig. 3:**
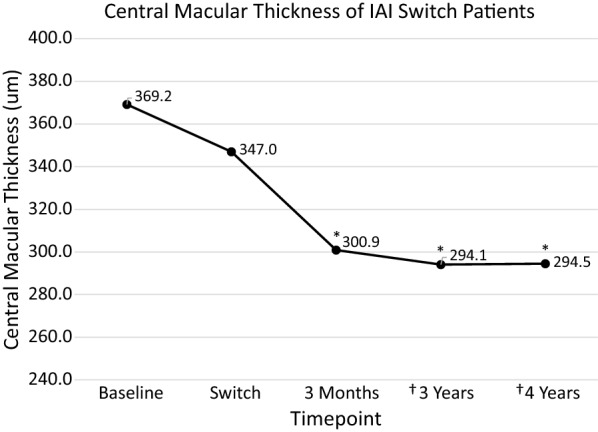
Central macular thickness of IAI switch patients. CMT decreased from baseline versus at the time of switch (p = 0.08). From the time of the switch to IAI to 3 months, CMT significantly decreased to 301 and was maintained to 48 months (*p < 0.05). In year 3, data is aggregated from 88 patients and in year 4, data is aggregated from 58 patients

The initial SD-OCT had a Central Macular Thickness (CMT) of 369 µm (Fig. 3), which improved to 347 µm at time of switch (p = 0.08), and further improved at 3 months to 301 µm (p < 0.0001). This CMT improvement was maintained over time at 3 and 4 years at 294 µm (p < 0.0001) and 295 µm (p < 0.0001), respectively, compared to baseline. A representative OCT is illustrated in Additional file1 Figure S1.

At baseline, 30 of 88 patients (34.1%) had 20/40 or better vision, while 46 of 88 (52.3%) had vision between 20/50 and 20/100, and 11 of 88 (12.5%) had 20/200 or worse (Table 2). Upon switching, 51 of 88 patients (58.6%) had 20/40 or better vision, 32 of 88 (36.8%) had vision between 20/50 and 20/100, and 5 of 88 (5.7%) had vision 20/200 or worse. At 3 months after the switch, 58 of 88 patients (65.9%) had 20/40 or better vision, 28 of 88 (31.8%) had vision between 20/50 and 20/100, and 2 of 88 (5.7%) had 20/200 vision or worse. Three years following the switch, 55 of 88 (62.5%) patients had 20/40 or better vision, 28 of 88 (31.8%) had vision between 20/50 and 20/100, and 5 of 88 (5.9%) had vision 20/200 or worse. At 4 years, 33 of 56 patients (58.9%) had 20/40 or better vision, 21 of 56 (27.5%) had vision between 20/50 and 20/100, and 2 of 56 (3.8%) had vision 20/200 or worse.

**Table 2:** Percentage of eyes with VA greater than 20/40, between 20/50 to 20/100, and 20/200 or worse

	Baseline	Switch	3 months	3 years	4 years
> 20/40	34.5%	58.6%	69.5%	62.5%	58.9%
20/50–20/100	52.9%	36.8%	31.8%	31.8%	37.5%
< 20/200	12.6%	5.7%	2.3%	5.7%	3.8%

Percentage of eyes with VA greater than 20/40, 20/50–20/100, and 20/200 or worse. From baseline to 3 months, the percentage of patients with 20/40 vision increased from 34.5 to 65.9%, while patients with 20/50–20/100 and 20/200 or worse vision steadily decreased. From 3 months to 4 years, the percentage of patients with 20/40 vision was in large part maintained from 65.9 to 58.9%, with the proportion of patients with 20/50–20/100 vision changing correspondingly

## Discussion

Anti-VEGF therapy has changed how retina specialists approach treatment of nAMD. Today, these agents are standard of care to maintain and improve vision in many patients. Seminal RCTs such as ANCHOR, MARINA, VIEW 1 and CATT, reported overall visual gains between 7.2 and 11.3 letters after 1–2 years of treatment [4–9]. However, over time much of those visual gains were lost, as reported in retrospective studies and the extension trials. [10, 11, 13–15].

There are different management approaches to treating nAMD over the short and long term. The approach of this retrospective study was to examine those patients that had a sub-optimal or non-response to other anti-VEGF agents and examine their long-term results, using a TES protocol, after switching therapy to aflibercept. In this study, the main indicator used to determine response to anti-VEGF therapy was visual acuity and the SD-OCT data.

Prior to switching to IAI, patients in this analysis were treated with other anti-VEGF agents for an average for 32 months. Although patients initially experienced improvement in VA and CMT from baseline, they were considered sub-optimal or non-responders. Their CMT improved from baseline, 369 µm to 347 µm, trending towards, but not statistically significant (p = 0.08) while on ranibizumab or bevacizumab. Once switched to aflibercept, SD-OCT data showed a statistically significant decrease in CMT at all subsequent time points with a consistently drier macula. The mean CMT decreased from 347 µm at the time of switch to 301 µm at 3 months. At 4 years, the CMT was maintained at 295 µm. Similarly, other switch studies demonstrated reduction in CMT [16–19]. In a retrospective switch study conducted by Cardoso et al., they evaluated patients who were switched from ranibizumab to aflibercept and the CMT decreased from baseline of 386.2 μm to 266.1 μm by year 3 [16]. Wykoff et al. published a 6-month prospective switch study to IAI with, CMT decreasing by 27.3 µm from time of switch [17]. In addition, Chatziralli et al. demonstrated that at 48 weeks, switching to IAI dosed every 8 weeks led to CMT decreasing from a baseline of 272 µm to 239 µm [18]. Spooner et al. conducted a switch study over 4 years with 49 patients, and CMT decreased an average 170.3 μm [19]. While those switch studies showed a similar pattern of CMT fluid reduction over time, the visual results varied, some of which may be explained by treatment protocols [16–19]. While this study employed a TES protocol, the average number of aflibercept injections ranged from 7.7 injections at year 1 to 6.6 injections at year 4. Chatziralli et al. demonstrated that switching to IAI dosed every 8 weeks at one year had decreased CMT, and stable VA [18]. In the other switch studies, there was a trend for visual decline over time. Cardoso et al. utilized individualized treatment including a combination of PRN, monthly dosing, and TAE. At the end of 3 years, patients experienced a steady deterioration of vision, with the average VA decreasing by 7 letters [16]. In Spooner’s study, utilizing a TAE variant, the patients lost all VA improvement, becoming statistically similar to the baseline, with 41% of patients improving 5 or more letters and 31% losing 5 or more letters [19].

In this study, we demonstrated that patients receiving consistent IAI therapy following the switch from other anti-VEGF agents were able to improve and maintain their vision gains from baseline, with an average VA gain of 8.1 letters, 3 years after the switch, with an average total treatment time of 5.7 years and 6.6 letters after an average treatment time of 6.7 years. It is of interest that the best vision was obtained, on average 3 months after switching to IAI, with an average increase in vision of 9.6 letters, but over time there was some visual decline.

In a previous study, we reported on the TES protocol for patients that received, on average a total of 63.7 injections, ranging from 8.1 to 9.6 per year. The overall mean visual gain was 8.7 letters at 8 years for the group, with 43% maintaining 20/40 vision or better [12].

In this study, a majority of patients were able to maintain driving vision, 20/40 or better in their treated eye. At 3 months after the switch, 65.9% had 20/40 or better vision, while at three years following the switch, 62.5% eyes had 20/40 or better vision. Even at 4 years after the switch to aflibercept, 58.9% maintained 20/40 or better vision.

While most patients in this study maintained good vision, 10.3% of patients lost 3 or more lines. This is similar to the VIEW four-year extension trial where only 8.2% of patients lost 15 or more letters [20]. This is in contradistinction to the Seven-Up study, where 34% of patients lost 15 letters (3 lines) or more [10] or the CATT-5 study where 28.6% of patients lost 3 or more lines of vision [11].

From the time of switch, patients had an increase of VA at three months that was statistically significant. After 3 and 4 years of therapy, the average visual gains were still maintained from baseline vision. The visual gains from baseline were likely maintained because patients were switched to IAI when there was a significant increase in fluid on SD-OCT but before there was a significant visual loss. The TES method proactively treats disease and therefore inhibits advancement or re-emergence of the disease process as previously described [3, 12]. This suggests that method of treatment may play a role in visual outcomes, since the current study used aflibercept but maintained improved visual and anatomical outcomes. It is possible that both consistent therapy prior to switching and performing the switch when there is still room for visual acuity improvement may play complementary roles in improving and maintaining visual outcomes over the long term. It is also possible that had the patients maintained their previous anti-VEGF agents, the increase in exudation, an indicator of increased disease activity, would have likely led to decreased vision in the future, since visual acuity often lags anatomic changes seen on SD-OCT [21].

Limitations of this study included its method of VA measurement, the nature of the study design, the sample size and probable “survivor” bias. The vision recorded was Snellen vision and was converted to ETDRS vision for purposes of analysis. Additionally, as a retrospective chart review, it included multiple physicians which may have led to limitations in the standardization of treatment regimen. There may have also been heterogeneity in treatment patterns including some variation in criteria used to switch patients to aflibercept. Also, this study did not examine the results of patients switched between the other anti-VEGF agents. There are reports that those switches may likewise have visual and anatomic improvement. There is most likely a survivor bias in this group. Since we only reported on patients that had anatomic worsening on SD-OCT and we utilized a TES protocol, those patients that developed geographic atrophy or fibrovascular scarring would have not met inclusion criteria. We previously reported on a cohort of patients that had stopped therapy, using the TES protocol. Baseline vision for that study was 20/125 and final vision at end of treatment was 20/125 with an average treatment time of 30 months [22]. This was due to a high number of patients, 46.6%, completing the TES protocol that had either central fibrovascular scarring (FV) or central GA. Patients with central GA or FV would not benefit from switching therapy. There were also no visual requirements to be included in this study. Patients with good vision were enrolled, which may have led to a ceiling effect, while patients with poor vision, any CNV lesion size, presence of RPE tears or hemorrhage were enrolled as well. The broad inclusion criteria may make the findings of this study more generalizable to a standard retina practice. Furthermore, the sample size was moderate, n = 88 at 3 years and n = 56 at 4 years. Prospective studies should be undertaken to compare patients that meet criteria to switch to other anti-VEGF agents and compare that to a group that did not switch anti-VEGF agents. However, the paucity of data regarding the long-term visual outcomes and effects of IAI on patients that switched from other anti-VEGF agents make the findings of this study relevant.

## Conclusions

Patients switched to IAI maintain vision over the long term. Patients treated on average for 5.75 years had a statistically significant improvement from baseline with an average visual gain of 8.1 letters after 3 years of continuous therapy and 6.9 letters after 4 years of continuous therapy after the switch to aflibercept. CMT significantly improved following the switch and was maintained over 4 years of active treatment. Patients that switched from other anti-VEGF agents to IAI, that are seen as suboptimal responders or treatment failures may have both improved visual outcomes over the short term and maintain visual gains over the long term, while improving their retinal anatomy on SD-OCT with consistent long term aflibercept therapy.

## Supplementary Information

**Additional file 1: Figure S1.** **a**–**d** *Representative SD-OCT of Study Patient.* **a** Patient presented with a new onset choroidal neovascular membrane with visual acuity of 20/80. **b** Patient was managed with four intravitreal bevacizumab injections and was felt to be a sub-optimal responder even though vision improved to 20/40. **c** After switching to intravitreal aflibercept, the patients vision improved to 20/30 at the three-month time period with decreasing central macular thickness. **d** Visual acuity is maintained at 20/30 after three years of consistent intravitreal aflibercept therapy with injections given approximately every 6–8 weeks with no central macular fluid

## Data Availability

The datasets used and/or analyzed during the current study are available from the corresponding author on reasonable request.

## Abbreviations

CMT: Central macular thickness
ETDRS: Early Treatment Diabetic Retinopathy Study
FV: Fibrovascular
GA: Geographic atrophy
IAI: Intravitreal aflibercept injections
nAMD: Neovascular age-related macular degeneration
PRN: Pro Re Nata
RPE: Retina pigment epithelium
SD-OCT: Spectral Domain Optical Coherence Tomography
TAE: Treat and extend
TES: Treat–extend–stop
VA: Visual acuity
VEGF: Vascular endothelial growth factor

## References

[1] Wykoff CC, Croft DE, Brown DM (2015). Prospective trial of treat-and-extend versus monthly dosing for neovascular agerelated macular degeneration: TREX-AMD 1-year results. Ophthalmology.

[2] Abedi F, Wickremasinghe S, Islam AF, Inglis KM, Guymer RH (2014). Anti-VEGF treatment in neovascular age-related macular degeneration: a treat-and-extend protocol over 2 years. Retina.

[3] Adrean  SD, Chaili S, Pirouz A (2018). Recurrence rate of choroidal neovascularization in neovascular age-related macular degeneration managed with a treat–extend–stop protocol. Ophthalmol Retina.

[4] CATT Research Group (2011). Ranibizumab and bevacizumab for neovascular age-related macular degeneratio. N Engl J Med.

[5] Martin DF, Maguire MG, Fine SL (2012). Ranibizumab and bevacizumab for treatment of neovascular age-related macular degeneration: two-year results. Ophthalmology.

[6] Rosenfeld PJ, Brown DM, Heier JS (2006). Ranibizumab for neovascular age-related macular degeneration. N Engl J Med.

[7] Heier JS, Brown DM, Chong V (2012). Intravitreal aflibercept (VEGF trap-eye) in wet age-related macular degeneration. Ophthalmology.

[8] Busbee BG, Ho AC, Brown DM (2013). Twelve-month efficacy and safety of 0.5 mg or 2.0 mg ranibizumab in patients with subfoveal neovascular age-related macular degeneration. Ophthalmology.

[9] Brown DM, Kaiser PK, Michels M (2006). Ranibizumab versus verteporfin for neovascular age-related macular degeneration. N Engl J Med.

[10] Rofagha S, Bhisitkul RB, Boyer DS (2013). Seven-year outcomes in ranibizumab-treated patients in ANCHOR, MARINA, and HORIZON: a multicenter cohort study (SEVEN-UP). Ophthalmology.

[11] Maguire MG, Martin DF, Ying GS (2016). Five-year outcomes with anti-vascular endothelial growth factor treatment of neovascular age-related macular degeneration: the comparison of age-related macular degeneration treatments trials. Ophthalmology.

[12] Adrean DS, Chaili S, Ramkumar H (2018). Consistent long-term therapy of neovascular age-related macular degeneration managed by 50 or more anti-VEGF injections using a treat–extend–stop protocol. Ophthalmology.

[13] Westborg I, Granstam E, Rosso A (2017). Treatment for neo- vascular age-related macular degeneration in Sweden: out- comes at seven years in the Swedish Macula Register. Acta Ophthalmol.

[14] Pedrosa AC, Sousa T, Pinheiro-Costa J (2017). Treatment of neovascular age-related macular degeneration with anti-VEGF agents: predictive factors of long-term visual outcomes. J Ophthalmol.

[15] Peden MC, Suñer IJ, Hammer ME (2015). Long-term outcomes in eyes receiving fixed-interval dosing of anti-vascular endo-thelial growth factor agents for wet age-related macular degeneration. Ophthalmology.

[16] Cardoso P, Pinheiro A (2017). Switch to aflibercept in the treatment of neovascular amd: long-term results. J Ophthalmol.

[17] Wykoff CC, Brown DM, Maldonado ME (2014). Aflibercept treatment for patients with exudative age-related macular degeneration who were incomplete responders to multiple ranibizumab injections (TURF trial). Br J Ophthalmol.

[18] Chatziralli I, Nicholson L, Vrizidou E (2016). Predictors of outcome in patients with neovascular age-related macular degeneration switched from Ranibizumab to 8-weekly aflibercept. Ophthalmology.

[19] Spooner K, Hong T, Nair R (2019). Long-term outcomes of switching to aflibercept for treatment-resistant neovascular age-related macular degeneration. Acta Ophthalmol.

[20] Kaiser PK, SInger M, Tolentino M (2017). Long-term safety and visual outcome of intravitreal aflibercept in neovascular age-related macular degeneration. Opththalmol Retina.

[21] Stoller GL, Kokame GT, Dreyer RF (2016). Patterns of early and delayed visual response to ranibizumab treatment for neovascular age-related macular degeneration. JAMA Ophthalmol.

[22] Adrean SD, Chaili S, Pirouz A, Grant S (2021). Results of patients with neovascular age-related macular degeneration managed by a treat–extend–stop protocol without recurrence. Graefes Arch Clin Exp Ophthalmol.

